# RNAi-Mediated Knock-Down of Arylamine *N*-acetyltransferase-1 Expression Induces E-cadherin Up-Regulation and Cell-Cell Contact Growth Inhibition

**DOI:** 10.1371/journal.pone.0017031

**Published:** 2011-02-09

**Authors:** Jacky M. Tiang, Neville J. Butcher, Carleen Cullinane, Patrick O. Humbert, Rodney F. Minchin

**Affiliations:** 1 School of Biomedical Sciences, University of Queensland, St. Lucia, Queensland, Australia; 2 Peter MacCallum Cancer Centre, East Melbourne, Victoria, Australia; Baylor College of Medicine, United States of America

## Abstract

Arylamine *N*-acetyltransferase-1 (NAT1) is an enzyme that catalyzes the biotransformation of arylamine and hydrazine substrates. It also has a role in the catabolism of the folate metabolite *p*-aminobenzoyl glutamate. Recent bioinformatics studies have correlated NAT1 expression with various cancer subtypes. However, a direct role for NAT1 in cell biology has not been established. In this study, we have knocked down NAT1 in the colon adenocarcinoma cell-line HT-29 and found a marked change in cell morphology that was accompanied by an increase in cell-cell contact growth inhibition and a loss of cell viability at confluence. NAT1 knock-down also led to attenuation in anchorage independent growth in soft agar. Loss of NAT1 led to the up-regulation of E-cadherin mRNA and protein levels. This change in E-cadherin was not attributed to RNAi off-target effects and was also observed in the prostate cancer cell-line 22Rv1. *In vivo*, NAT1 knock-down cells grew with a longer doubling time compared to cells stably transfected with a scrambled RNAi or to parental HT-29 cells. This study has shown that NAT1 affects cell growth and morphology. In addition, it suggests that NAT1 may be a novel drug target for cancer therapeutics.

## Introduction

Human arylamine *N*-acetyltransferase-1 (NAT1; EC 2.3.1.5) is a well characterized enzyme that catalyzes the transfer of an acetyl group from acetyl coenzyme A to the amino group of arylamine and/or hydrazine compounds [1]. NAT1 is genetically polymorphic and different alleles express proteins with altered catalytic activity [2]. Although NAT1 has a widespread tissue distribution, the folate catabolite *p*-aminobenzyolglutamate is the only known endogenous substrate [3]. NAT1 gene expression involves 2 promoters that generate at least 9 different transcripts, all with an identical protein coding sequence. The NAT1 gene is inducible with androgens [4] and stability of the NAT1 protein is affected by substrates, which enhance poly-ubiquitination and degradation [5], [6].

In human breast cancer samples, NAT1 mRNA levels have been shown to cluster with a group of genes that included the estrogen receptor, being highest in luminal-type carcinomas and lowest in basal-like carcinomas [7], [8], [9]. The selective expression of NAT1 in tumor subtypes is not confined to breast cancer. In a gene profiling study, Lapointe and coworkers identified 3 separate prostate cancer subtypes, each with markedly different levels of NAT1 expression [10]. NAT1 mRNA levels also appear to be up-regulated in p53 wild-type tumors compared to p53 loss-of-function tumors [11].

When NAT1 was over-expressed in the immortalized breast epithelial cell line HB4a, the resulting cells showed an increased proliferative capacity and the ability to grow in low serum conditions [8]. Moreover, the cells showed enhanced resistance to the cytotoxic drug etoposide. These studies implied that NAT1 may play a role in cell growth and survival, although no underlying mechanism has been proposed. The purpose of the present study was to investigate the effects of altered NAT1 expression on cell proliferation and to characterize any changes in phenotype associated with NAT1 activity. Previously, we have generated several cell lines that over-express NAT1 [5]. However, we observed no changes in proliferation or cell morphology. Here, we report the effects of silencing NAT1 using shRNA. For these studies, we chose the well-characterized human colon adenocarcinoma cell line HT-29 because these cells carry only one copy of the NAT1 gene due to a deletion at 8p21-22 [12]. Normally, HT-29 cells express NAT1 at levels approximately half that seen in other cell lines [13]. These cells also carry a truncated APC gene [14] and mutations in the p53 gene [15]. Using HT-29 cells stably expressing shRNA targeting NAT1 mRNA, we have compared the growth kinetics and phenotypic changes associated with altered NAT1 expression.

## Materials and Methods

### Cell culture

The HT-29 colon adenocarcinoma cell line was obtained from the American Type Culture Collection (Manassas, VA, USA). Cells were cultured in RPMI 1640 supplemented with 5% fetal calf serum and penicillin/streptomycin and maintained at 37°C in a humidified atmosphere of 5% CO_2_ in air.

### Cell transfection

The Suresilencing™ shRNA plasmid targeting the human NAT1 open reading frame was purchased from SuperArray, Bioscience Corporation, MD, USA. Transfection was performed using Lipofectamine2000 (Invitrogen, Carlsbad, CA, USA) according to the manufacturer's protocol. The shRNA in controls is a scrambled sequence that does not have homology to any human gene. Stable cell lines were selected with 1 mg/ml G418. To generate shRNA 3.2+NAT1 cells, linearized pEF-Neo plasmid containing NAT1 was co-transfected with pTK-HYG and stable cell lines were selected with 0.5 mg/ml Hygromycin.

### NAT1 activity assay

Cells were washed with PBS, resuspended in lysis buffer (20 mM Tris, pH 7.4, 1 mM EDTA, and 1 mM DTT) and lysed by sonication. Cell lysates were then centrifuged at 14,000×*g*, 4°C for 10 min and the supernatant assayed for NAT1 activity by high performance liquid chromatography as previously described [6]. Protein concentrations were determined by the method of Bradford [16].

### Cell proliferation assay

Cells were plated at a density of 5,000 cells/well in 96-well plates. After 24 h, growth rate was measured using the CellTiter96® AQueous Non-Radioactive Cell Proliferation Assay kit (Promega, Madison, WI, USA) over 5–6 days following the manufacturer's instructions. The conversion of MTS into formazan was measured at 540 nm with a micro-plate reader (Bio-Rad, Hercules, CA, USA).

### Cell viability

Plated cells at day 4 were stained with 1 µg/ml propidium iodide on ice for 20 min and analyzed by FACSCanto™ flow cytometry. The dead cells population was determined as the percentage of propidium iodide stained cells.

### Transmission Electron Microscopy

HT-29 cells were grown on dishes and fixed with 3% glutaraldehyde (ProSciTech, Townsville, Australia) in 0.1 M phosphate buffer and then post-fixed with 1% osmium tetroxide (ProSciTech). Following dehydration in an ascending series of ethanol, specimens were embedded in a thin layer of LX 112 resin (Ladd Research, Williston, Vermont, USA). The resin was polymerized at 60°C for 48 h in an oven before ultrathin sections were stained with uranyl acetate (SPI, West Chester, Pennsylvania, USA) and lead citrate prior to imaging. Samples were cut to reveal both transverse and longitudinal sections. Transmission electron microscopy images were taken using a JEOL 1010 (JEOL, Tokyo, Japan) fitted with a SIS Megaview III digital camera and operated at 80 kV.

### Anchorage-independent colony formation

Cells were suspended in 2 ml complete media containing 0.3% agar (Sigma-aldrich, St. Louis, MO, USA) and seeded in to wells (3,000 cells/well) layered with 0.6% base agar. 10–14 days after seeding, colonies were stained with 0.005% crystal violet, photographed and counted using Quantity One Software (Bio-Rad). The size of colonies was determined using Adobe Photoshop 7.

### Western blot analysis

Cells were lysed in SDS reducing buffer and electrophoresed on 12% polyacrylamide gels, transferred to nitrocellulose membranes, and immunoblotted overnight at 4°C with either anti-E-cadherin (HECD-1, Abcam, Cambridge, UK), anti-SNAIL (sc-28199, Santa Cruz Biotechnology, Santa Cruz, CA, USA), anti-SLUG (ab27568, Abcam, Cambridge, UK), or anti-Twist (sc-15393, Santa Cruz Biotechnology). Membranes were washed and then incubated with horseradish peroxidase conjugated secondary antibodies for 1 h, washed, and then levels of protein detected using Immun-Star™ HRP Chemiluminescent kit (Bio-Rad). Tubulin immunoblot using DM1A antibody (Calbiochem, Darmstadt, Germany) was used as the loading control.

### Immunocytochemistry

Cells that had been grown on coverslips were chilled on ice for 15 min, washed with cold PBS, and then fixed with 2% formaldehyde containing 0.1% Triton-X on ice for 30 min. Cells were blocked with 1% BSA at room temperature for 1 h. For F-actin staining, cells were incubated with Alexa Fluor 647 conjugated phalloidin (Molecular Probes, USA) for 1 h and samples were mounted using Vectorshield® mounting medium with DAPI (Vector Laboratories, Inc., Burlingame, CA, USA). For E-cadherin immunocytochemistry, after blocking with BSA, cells were blotted with E-cadherin antibody (HECD-1, Abcam) overnight at 4°C. Cells were then washed and probed with Alexa Fluor 488® conjugated secondary antibody (Molecular Probes) for 1 h prior to mounting with Vectorshield® mounting medium with DAPI (Vector Laboratories, Inc.,). Slides were visualized using an Olympus BX61 confocal microscope.

### Extraction of total RNA and cDNA synthesis

Exponentially growing cells were washed with PBS and total RNA was extracted using TRIzol reagent (Invitrogen) according to manufacturer's instructions. RNA was resuspended in RNase-free water and reverse transcribed using Superscript II Reverse Transcriptase (Invitrogen) and oligo(dT)_15_ primer (Promega).

### Quantification of mRNA by real-time PCR

Expression levels of mRNA were determined using an iCycler IQ Real-time PCR Detection System (Bio-Rad). For endogenous NAT1 mRNA, first-strand cDNA was amplified using forward primer 5′- TCTAGTTCCTGGTT-GCCGGCTGAAATAACC-3′ and reverse primer 5′-CTCAAAGGGAACCG-CGGGGATCTGGTGTTG-3′. For E-cadherin mRNA expression, forward primer 5′-CAGCATCACTGGCC-AAGGAGCTGA-3′ and reverse primer 5′-GACCACACTGAT-GACTCCTGTGTTCC-3′ were used. β-actin (forward primer: 5′-TCACCCACACTGT-GCCCATCTACGA-3′, reverse primer 5′-CAGCGGAACCGCT-CATTGCCAATGG-3′) was used for normalization. Reactions contained IQ Supermix (Bio-Rad), 6 pmol of each primer, and 2 µl of cDNA in a total volume of 25 µl. PCR conditions were 95°C for 90 s, followed by 40 cycles of 95°C for 15 s, 60°C (55°C for β-actin) for 20 s, and 72°C for 30 s. A melting curve was obtained to verify specificity of the PCR.

### *In vivo* tumor growth

All animal procedures were performed with approval from the Peter MacCallum Cancer Centre animal experimentation ethics committee. Control 4 or shRNA cells (3×10^6^) in 50% Matrigel (Becton Dickinson) were implanted subcutaneously into the right flank of 6–8 week old female Balb/c nude mice (Animal Resources Centre, Western Australia). Once tumors were established, they were measured twice weekly using electronic calipers and tumor volume was calculated according to the formula: length/2× width^2^. On harvest, the tumors were divided in half and one sample was snap frozen in liquid nitrogen while the other sample was fixed in formalin and embedded in paraffin. The snap frozen samples were homogenized for assaying NAT1 activity or sonicated in TRIzol reagent for RNA extraction. H&E and immunochemical staining were performed on the paraffin embedded samples.

### Immunohistochemistry

Staining for Ki-67 was on 6 µm thick formalin-fixed, paraffin-embedded tumor sections. After de-waxing, antigen retrieval was performed with a 10 nM citrate buffer (pH 6.0) in a decloaking chamber for 5 min at 125°C. Endogenous peroxidase activity was inhibited using methanol and hydrogen peroxide. Immunohistochemical staining was carried out using Vectastain ABC kit (Vector Laboratories, Inc.). A MIB-1 monoclonal antibody (Dako, Carpenteria, CA, USA) at a 1∶100 dilution was used to probe human ki-67 for 2 h at room temperature. Sites of peroxidase activity were detected using 3,3-diaminobenzidine/hydrogen peroxide. Counterstaining was performed with hematoxylin.

### Data analysis

All experiments were performed in triplicate. Data are expressed as mean ± SEM. Statistical comparisons between different groups were assessed by Student's *t* test or one-way ANOVA assuming significance at p<0.05 using Prism 4 (GraphPad Software, Inc., San Diego, CA, USA). Tumor growth rates were compared by two-way ANOVA.

## Results

### NAT1 knock-down with shRNA altered cell growth and survival

**Figure 1:**
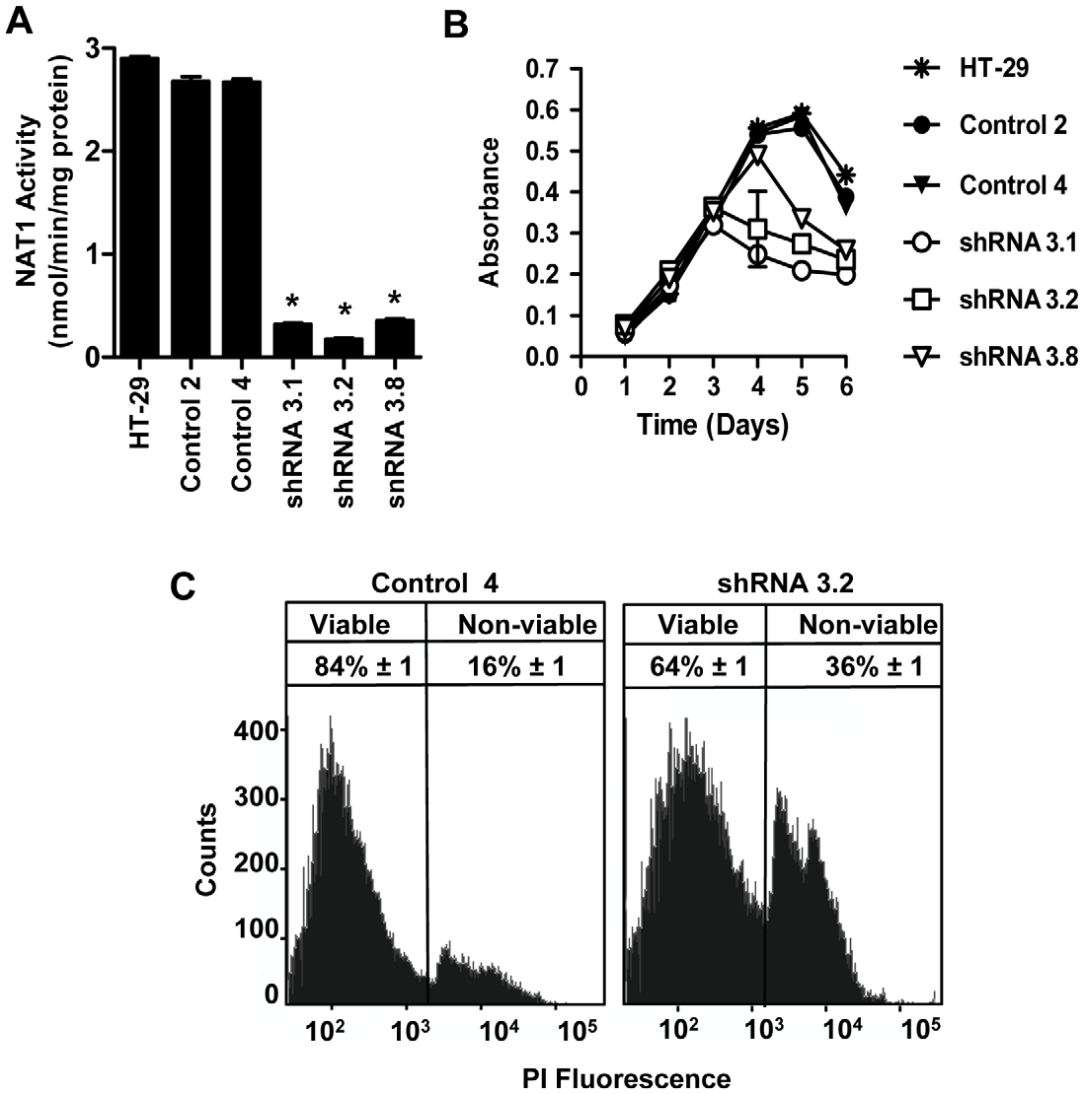
NAT1 knock-down alters cell growth and survival in HT-29 cells. (**A**) NAT1 activity in HT-29 cells and cells stably transfected with a scrambled shRNA (Control 2 and Control 4) or NAT1-directed shRNA (shRNA 3.1, shRNA 3.2 and shRNA 3.8). Results are presented as mean ± SEM (*n* = 3). Asterisks denote significant difference compared to HT-29 (p<0.05). (**B**) Growth of HT-29 shRNA clones. All clones were grown in RPMI 1640 supplemented with 5% FCS. Each point is the mean ± SEM (*n* = 3). (**C**) Viability of control cells (clone Control 4) and knock-down cells (clone shRNA 3.2) at day 4. Non-viable cells were stained with propidium iodide (PI) and quantified by flow cytometry. The reported percent viable and non-viable cells are shown as the mean ± SEM (*n* = 3).

HT-29 cells were stably transfected with a shRNA expression vector targeting NAT1 mRNA. Three independent clones were selected and NAT1 activity was determined. In all three clones, NAT1 activity was decreased by greater than 85% compared to the non-transfected parental HT-29 cells or control cells transfected with a scrambled shRNA sequence (Figure 1A). Proliferation of the NAT1 knock-down cells with time was similar to the control cells or non-transfected HT-29 cells during exponential growth (Figure 1B). However, the NAT1 knock-down cells reached a saturation density earlier than the control cells. Following confluence at day 3, the number of NAT1 knock-down cells in culture declined and this was associated with an increase in cell death (Figure 1C). For the control cells, saturation density was not reached until approximately day 5.

### NAT1 knock-down with shRNA led to morphological changes

**Figure 2:**
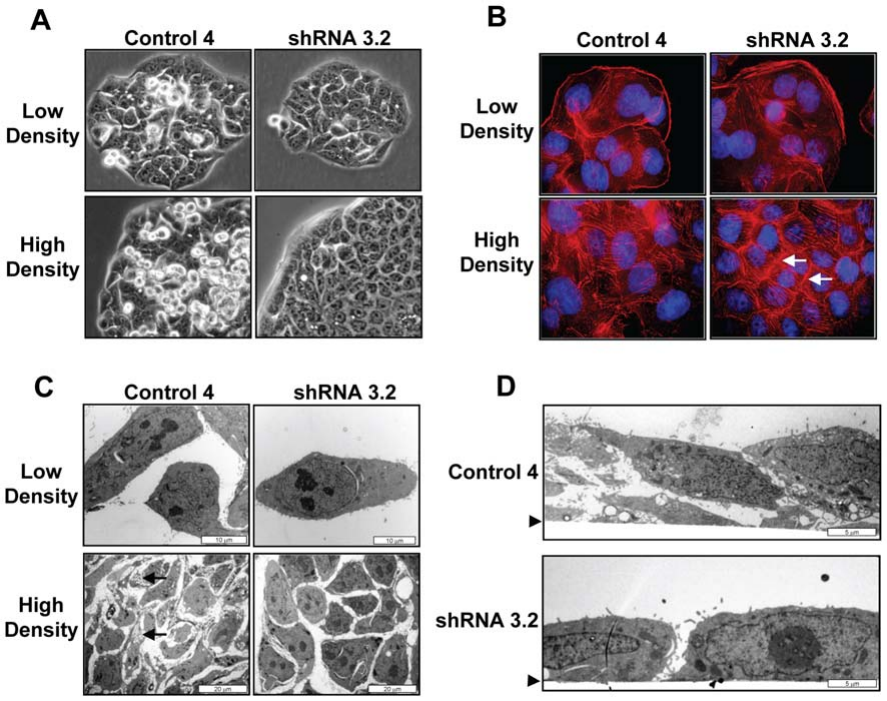
The effects of NAT1 knock-down on cell morphology. (**A**) Light microscopy of control (Control 4) and knock-down (shRNA 3.2) cells at low density (upper panels) and high density (lower panels). (**B**) Confocal microscopy of actin polymerization in cells at low (upper panels) and high (lower panels) density. Actin was stained with Alexa Fluor 647 conjugated phalloidin (red) and the cell nuclei were stained with DAPI (blue). White arrows show sub-cortical actin associated with sites of cell-cell contact (lower right panel). (**C**) Transmission electron microscopy of control and NAT1 knock-down cells at low and high density. Black arrows show vacuoles. (**D**) Longitudinal sections of control and NAT1 knock-down cells by transmission electron microscopy at high density. Arrowheads indicate the bottom of the culture flask.

At sub-confluent density, both the control and NAT1 knock-down cells grew as monolayers and demonstrated a typical epithelial morphology (Figure 2A, upper panels). Actin filaments were generally unorganized and mostly associated with the sub-cortical cytoskeleton (Figure 2B, upper panels). Transmission electron microscopy also showed a similar morphology between the control and knock-down cells at low density (Figure 2C, upper panels). At high density, control cells exhibited multi-layer growth characteristics (Figure 2A, lower panels), which was also evident by transmission electron microscopy of confluent cells processed for viewing in the longitudinal plane (Figure 2D, upper panel). Unlike cells at low density, actin was mostly associated with intracellular stress fibers at high density (Figure 2B, lower panels). The control cells were highly vacuolated with vesicles that accumulated towards one end of the cell (Figure 2C, lower left panel, arrows), possibly indicative of cell polarization [17]. By contrast, the NAT1 knock-down cells maintained a monolayer at high density (Figure 2A and D, lower panels). The cells were larger and more cobble-shaped (Figure 2C, lower right panel). Actin stress fibers were seen in all cells and polymerized actin remained associated with the sub-cortical cytoskeleton and near sites of cell-cell contact (Figure 2B, lower right panel, arrows). Under transmission electron microscopy, very few intracellular vacuoles were seen in the knock-down cells (Figure 2C, lower right panel). These results indicate that loss of NAT1 is associated with a marked change in cell morphology.

### Loss of NAT1 inhibited anchorage-independent growth and up-regulated E-cadherin protein expression

**Figure 3:**
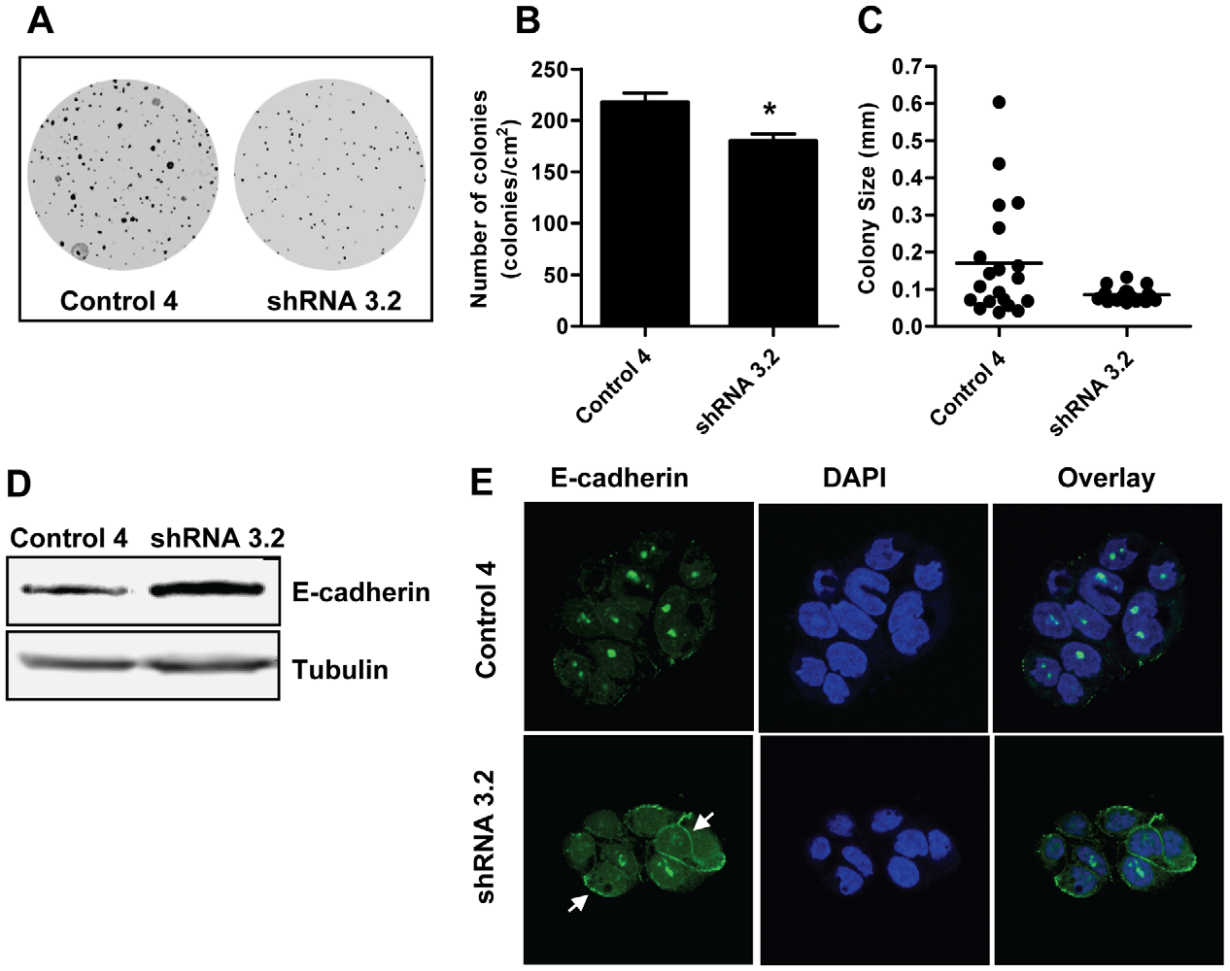
NAT1 knock-down impedes anchorage-independent growth in HT-29 cells and up-regulates E-cadherin protein expression. (**A**) Colony formation of control (Control 4) and NAT1 knock-down cells (shRNA 3.2) in soft agar. Cells were seeded at 1,500 cells/ml on 0.6% agar and grown for 10-14 days. Colonies were visualized with 0.005% crystal violet. (**B**) Colony numbers were determined from 3 independent soft agar plates and are presented as mean ± SEM. Asterisk denotes significant difference compared to Control 4 (p<0.05). (**C**) Colony size (diameter) distribution for control and knock-down cells. (**D**) Western blot analysis of E-cadherin in control and NAT1 knock-down cells. Immunoblotting was performed with E-cadherin antibody and tubulin was probed as the loading control. The blot is representative of three independent experiments. (**E**) Immunocytochemistry of E-cadherin expression in control and NAT1 knock-down cells. The increased E-cadherin protein expression was localized to the cell membrane (white arrows) in the NAT1 knock-down cells compared to control cells. E-cadherin was detected with anti-E-cadherin antibody followed by Alexa Fluor 488-conjugated secondary antibody (green) and visualized under a fluorescence confocal microscope. The nuclei were stained with DAPI (blue).

The ability for HT-29 cells to grow as multilayer cultures has been associated with anchorage-independent growth [18]. To test if NAT1 knock-down affected this, we examined colony formation of control and knock-down cells in soft agar (Figure 3A). Compared to cells treated with shRNA, the control cells formed large colonies clearly visible to the eye. Colony count showed a significantly greater number of colonies for the control cells (Figure 3B), although the difference was only approximately 20%. A much greater difference was seen when the size of the colonies was quantified (Figure 3C). The control cells showed a wide distribution in diameter ranging from 0.04 to 0.6 mm (0.17±0.03 mm) whereas the diameter of the NAT1 knock-down cell colonies was much smaller (0.08±0.004 mm), as was the range (0.06–0.13 mm). These results, along with the lower saturation density seen in Figure 1B, are consistent with cell-cell contact inhibition of growth.

E-cadherin is a key protein involved in cell adhesion and reportedly dictates responsiveness to contact inhibition in many cancer cell-types [19]. We therefore examined the expression of E-cadherin following NAT1 knock-down. E-cadherin protein expression increased in the knock-down cells compared to the control cells (Figure 3D). The increased E-cadherin expression was primarily associated with the plasma membrane in the knock-down cells (Figure 3E). These results show that NAT1 knock-down restores cell contact inhibition in HT-29 cells, which could explain the lower saturation density seen in the growth curves (Figure 1B). The restoration of contact inhibition is possibly due to increased E-cadherin expression.

### Loss of NAT1 up-regulated E-cadherin mRNA

**Figure 4:**
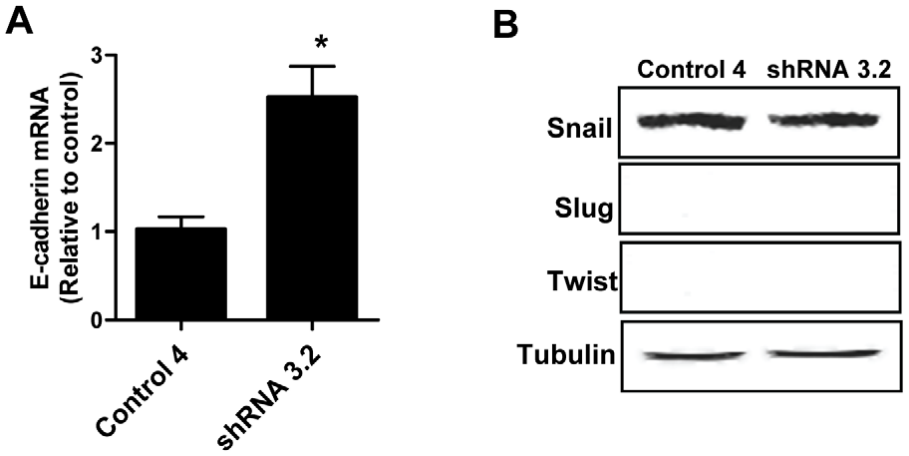
NAT1 knock-down up-regulates E-cadherin mRNA and alters DNA methylation status in the cell. (**A**) E-cadherin mRNA levels in NAT1 knock-down cells. Each value is mean ± SEM (*n* = 3). The asterisk denotes significant difference from control (p<0.05). (**B**) Western blot analysis of E-cadherin suppressor proteins in control and NAT1 knock-down cells. Twist and Slug were not detected in either cell-line and there was no difference in the expression of Snail. Tubulin was probed as the loading control.

Quantification of E-cadherin mRNA revealed that up-regulation of E-cadherin protein following NAT1 knock-down was accompanied by an increase in E-cadherin mRNA (Figure 4A), which was 2.5-fold higher in the shRNA3.2 cells. We measured the expression level of the E-cadherin transcriptional repressors Snail, Slug, and Twist (Figure 4B). There was no evidence of Slug or Twist expression in either cell line, and Snail showed no change following NAT1 knock-down. The results suggest that the increased E-cadherin mRNA expression is independent of the transcription repressors. E-cadherin can be regulated at transcriptional and/or epigenetic levels [20]. Further studies are required to determine if the change in E-cadherin expression is due to altered DNA methylation status of the cells.

### The up-regulation of E-cadherin by NAT1 shRNA was not due to off-target effects

**Figure 5:**
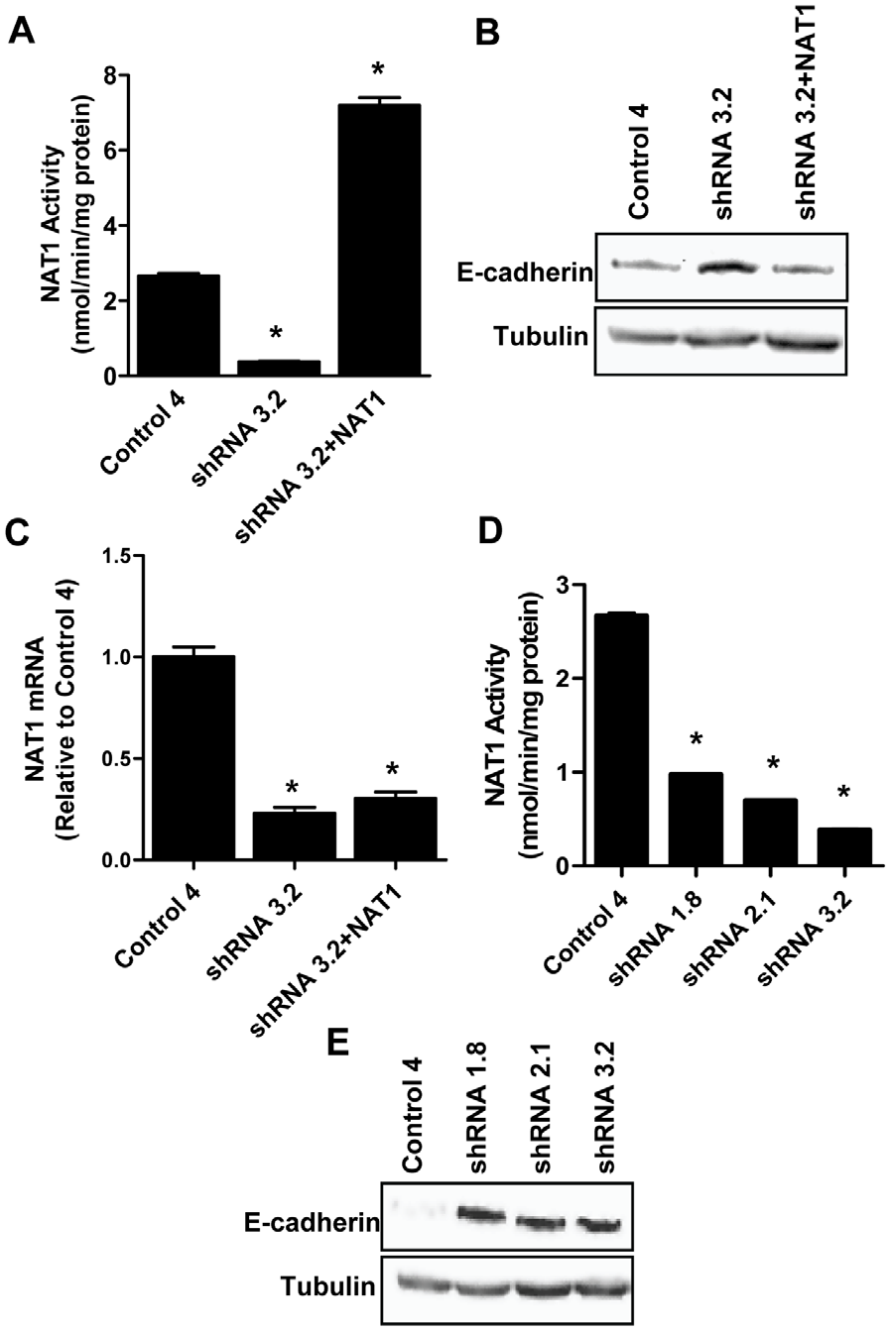
The effects of NAT1 knock-down by shRNA are not due to off-target effects. (**A**) NAT1 was reintroduced into the shRNA 3.2 cells (shRNA 3.2+NAT1) and NAT1 activity measured. Each value is mean ± SEM (*n* = 3). The asterisk denotes a significant difference compared to Control 4 (p<0.05). (**B**) Control 4, shRNA3.2, and shRNA3.2+NAT1 cell lysates were immunoblotted for E-cadherin. Tubulin was probed as the loading control. The blot is representative of 3 independent experiments. (**C**) NAT1 mRNA levels were quantified by real-time PCR using primers that detect endogenous NAT1 mRNA but not NAT1 mRNA derived from the exogenous plasmid. The data are presented as mean ± SEM (*n* = 3). Results were normalized to β-actin levels and then expressed relative to Control 4 mRNA. Asterisks denote significant difference compared to Control 4 (p<0.05). (**D**) Different independent non-overlapping shRNA targeting the NAT1 open reading frame were used to knock-down NAT1 and then NAT1 activity was assessed for each of the different clones. Each value is mean ± SEM (*n* = 3). Asterisks denote significant difference compared to Control 4 (p<0.05). (**E**) The effect of NAT1 knock-down by the different shRNA sequences on E-cadherin protein expression was determined by immunoblotting. Tubulin was probed as the loading control.

While shRNA-mediated gene silencing is highly sequence specific, off-target effects are not uncommon [21]. To determine if the effects of the NAT1 shRNA were due to the targeting of other unidentified transcripts, two experiments were performed. Firstly, NAT1 was reintroduced into the NAT1 knock-down cells using a plasmid with the NAT1 gene under the control of the CMV promoter to induce strong gene expression. NAT1 activity was restored to a level greater than that seen in the parental HT-29 cells (Figure 5A). Rescue of the NAT1 reversed the up-regulation of E-cadherin in the knock-down cells (Figure 5B). To ensure that the re-introduction of NAT1 did not switch off expression of the shRNA, endogenous NAT1 mRNA was quantified by amplification of exon 4, an exon located in the 5′UTR of NAT1 mRNA but is absent in the plasmid used to rescue NAT1 activity. Endogenous NAT1 mRNA remained low and similar to that in the knock-down cells following reintroduction of the gene (Figure 5C), indicating that these cells were actively expressing both the shRNA and exogenously introduced NAT1.

In a second experiment, two additional non-overlapping independent shRNA sequences targeting the NAT1 open reading frame were used to silence NAT1 expression. All three shRNA sequences significantly decreased NAT1 activity (Figure 5D). In addition, all three sequences increased E-cadherin expression (Figure 5E). Taken together, these results indicate that the effects of NAT1 knock-down on E-cadherin expression are unlikely to be due to off-target effects.

### E-cadherin up-regulation was not confined to HT-29 cells

**Figure 6:**
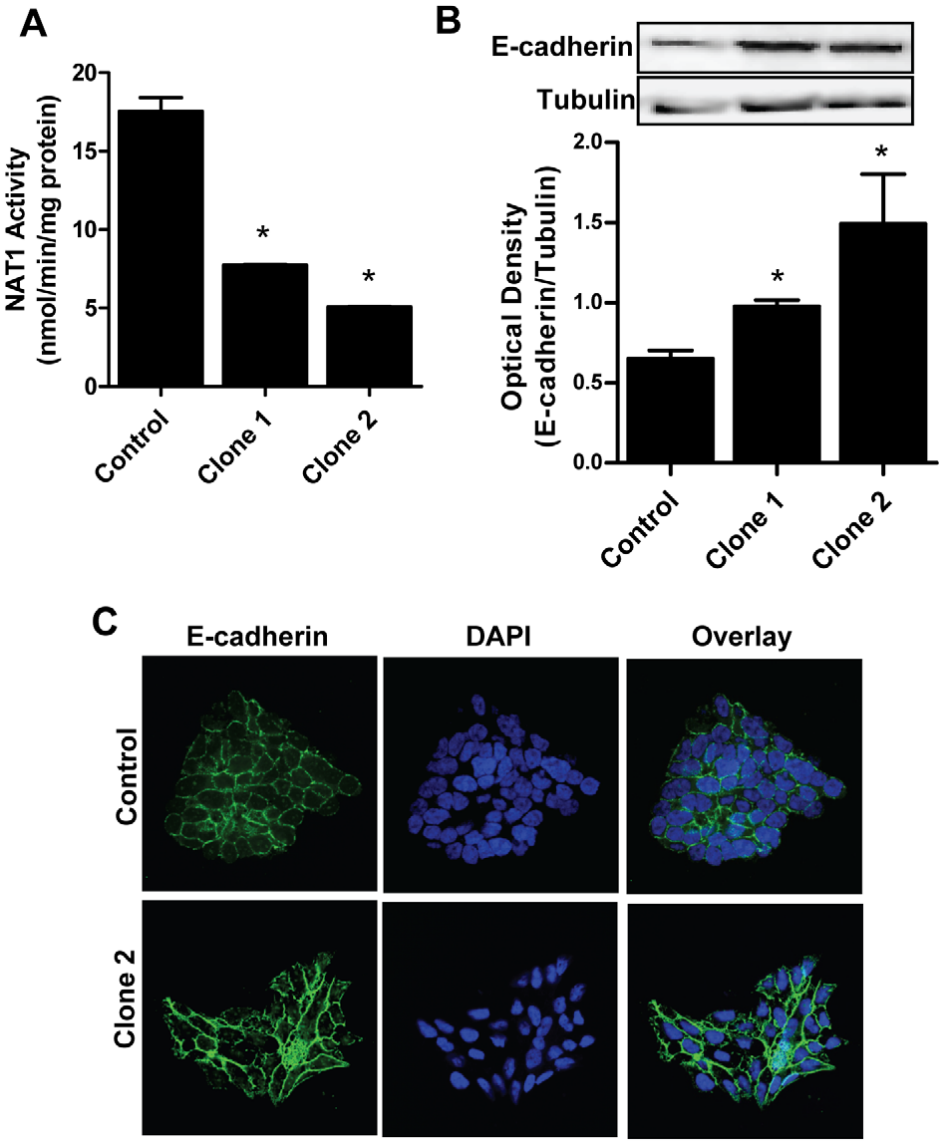
Up-regulation of E-cadherin following NAT1 knock-down is not cell-type specific. (**A**) 22Rv1 cells were stably transfected with the NAT1-directed shRNA construct (Clones 1 and 2) and knock-down determined by NAT1 activity assay. Results are presented as mean ± SEM (*n* = 3). Asterisks denote significant difference compared to the control (p<0.05). (**B**) Western blot analysis of E-cadherin protein in 22Rv1 control and NAT1 knock-down cells. Immunoblotting was performed with E-cadherin antibody and tubulin was probed as the loading control. The blot is representative of 3 independent experiments. Immunoblots were quantified by densitometry after normalization to tubulin. Results are presented as means ± SEM (*n* = 3). A representative blot is shown above the graph. Asterisks denote significant difference compared to control (*P*<0.05). (**C**) Immunocytochemistry of E-cadherin expression in 22Rv1 control and NAT1 knock-down cells. E-cadherin was detected with anti-E-cadherin antibody followed by Alexa Fluor 488-conjugated secondary antibody (green) and visualized under a fluorescence microscope. The nuclei were stained with DAPI (blue).

HT-29 cells are derived from a human colon adenocarcinoma that has a large deletion at 8p21, the site of the NAT1 gene. In addition, it has mutant p53 and truncated APC genes. To ensure our observations following NAT1 silencing were independent of the cell line, we generated stably transfected shRNA lines using the prostate cell line 22Rv1. These cells are also epithelial but have normal functioning p53 and APC. We selected 2 clones that showed a significant decrease in NAT1 activity (Figure 6A). In each clone, E-cadherin expression was increased compared to control cells stably transfected with a scrambled sequence (Figure 6B). Up-regulation of E-cadherin was also observed in these cells by immunocytochemistry where the majority of the protein was associated with the plasma membrane (Figure 6C). These results showed that up-regulation of E-cadherin following NAT1 knock-down was not confined to HT-29 cells.

### NAT1 knock-down affected tumor growth *in vivo*

**Figure 7:**
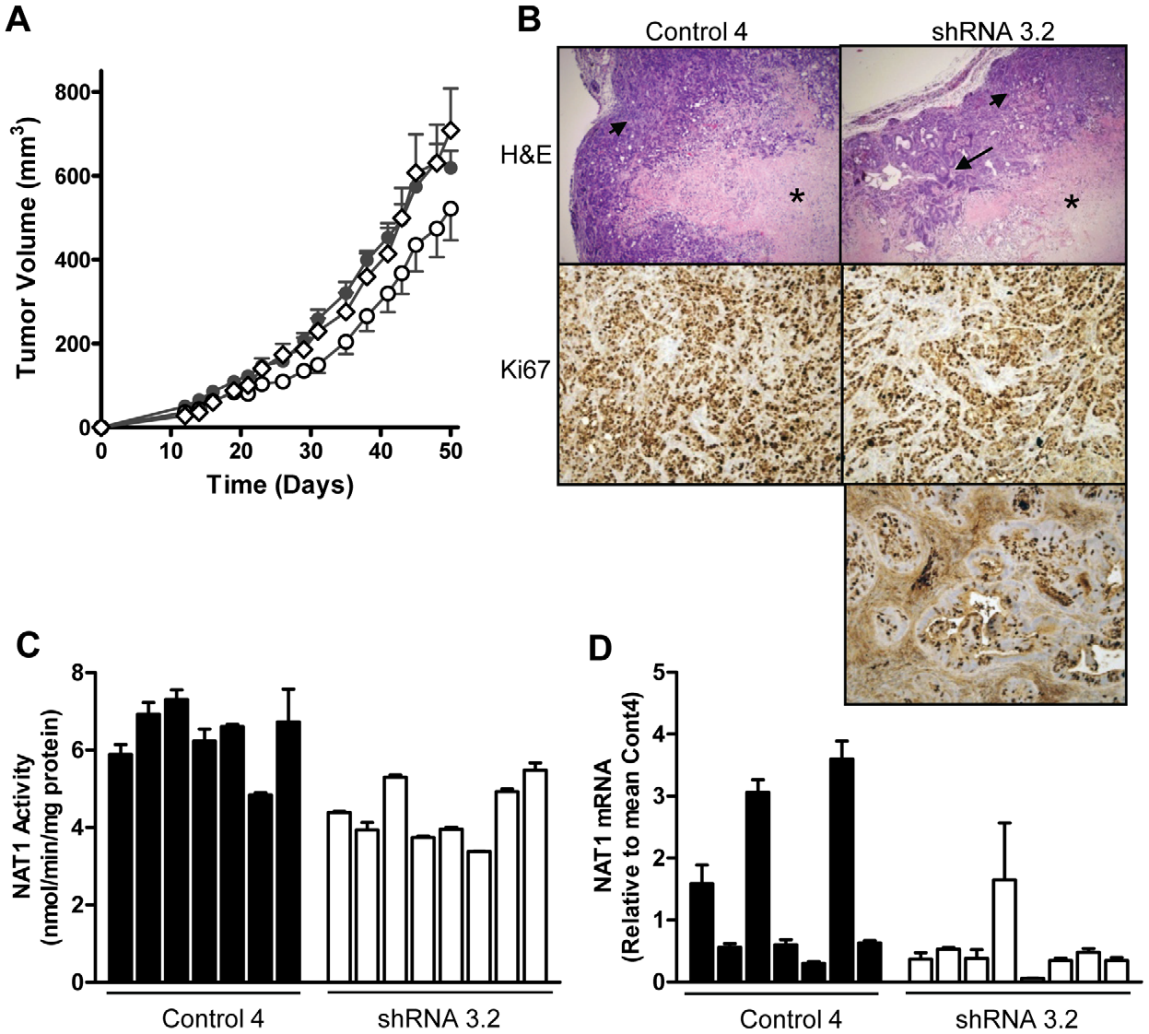
Growth of control and NAT1 knock-down cells *in vivo*. (**A**) Female Balb/c nude mice were injected subcutaneously with parental HT-29 cells (◊), Control 4 cells (•), or shRNA3.2 cells (○). Tumor volume was determined as outlined in the Methods. Results are mean ± SEM (*n* = 7–8). (**B**) Representative H&E staining of tumors (upper panels) from Control 4 (left panel) or shRNA 3.2 (right panel) showing large areas of necrosis (*) along with loosely organized columnar cells (small arrows). In the tumors derived from the shRNA 3.2 cells, more organized glandular structures were evident (large arrow). Representative immunohistochemistry for Ki67 in Control 4 and shRNA 3.2 tumors is shown in the middle and lower panels. (**C**) NAT1 activity in individual tumors from Control 4 and shRNA3.2 tumor tissue. The results are mean ± SEM (*n* = 3). (**D**) Real-time PCR of human NAT1 mRNA in individual tumors from Control 4 and shRNA3.2 tumor tissues. Results were normalized to β-actin and then expressed relative to Control 4. The results are mean ± SEM (*n* = 3).

To evaluate the effect of NAT1 knock-down on tumor growth *in vivo*, nude mice were injected subcutaneously with parental HT-29, Control 4 or shRNA 3.2 cells and tumor development monitored over 50 days (Figure 7A). Both the HT-29 and the control cells grew at the same rate *in vivo* with an estimated doubling time of 10.7 days (95% CI  = 10.6–11.0). Two-way analysis of variance showed that the shRNA growth curve was significantly different to either the parent HT-29 cells or the Control 4 cells (p<0.05). This was due to a modest but significant increase in the doubling time of the shRNA cells to 12.1 days (95% CI  = 11.9–12.2). At the end of the study, tumor tissue was collected for histological analysis and enzyme activity measurements. Histologically, the adenocarcinomas from the Control 4 cells contained large areas of necrosis surrounded by neoplastic columnar cells with a loose organization (Figure 7B, top left panel). The shRNA 3.2 cells formed similar adenocarcinomas containing mostly necrotic areas surrounded by columnar cells. However, these tumors also contained well-organized glandular bodies suggestive of a more differentiated state (Figure 7B, top right panel). Immunohistochemistry of the neoplastic columnar cells using an anti-Ki67 antibody to detect proliferating cells showed little difference between the Control 4 tumors and the shRNA tumors (Figure 7B, middle panels). However, the glandular tissue in the shRNA tumors contained large tracts of non-proliferative cells (Figure 7B, lower panel).

NAT1 activity was 30% lower in the NAT1 knock-down tumors compared to the control tumors (4.39±0.27 and 6.34±0.31 nmol/min/mg protein, respectively; Figure 7C), which is less than the 85% decrease seen in the shRNA 3.2 cells in culture (Figure 1A). This difference between the *in vitro* and *in vivo* NAT1 activities could be due to the presence of mouse cell infiltrates in the tumor samples, which express arylamine *N*-acetyltransferase. However, the H&E staining and the immunohistochemistry, which used a human-specific anti-Ki67 antibody, indicated that there was little evidence for excessive infiltration of mouse cells in the xenografts. An alternative explanation for the loss of NAT1 knock-down *in vivo* is the lack of selection with G418 during the *in vivo* study. If this were the case, then the level of human NAT1 mRNA in the knock-down tumors would approach that in the control tumors. We extracted total RNA from the tumor tissue and quantified human NAT1 mRNA using specific primers that did not amplify the mouse NAT homolog (Figure 7D). The relative amount of human NAT1 mRNA was variable between samples and was not significantly different between the 2 groups. (p = 0.08). These results suggest that the knock-down of NAT1 was not maintained in HT-29 cells grown *in vivo*, where there was no G418 selection. Nevertheless, the growth of the NAT1 knock-down cells was attenuated, indicating that NAT1 may have a role in tumor cell growth and/or survival *in vivo*.

## Discussion

When NAT1 was found to be involved in the metabolism of the folate catabolite *p*-aminobenzoylglutamate, it was hypothesized that the protein may have an important endogenous function in addition to acetylation of xenobiotics [3]. Mouse gene knockout models of NAT1 (*Nat2* or *muNat2*) have been developed independently in 2 laboratories [22], [23]. However, while acetylation of *p*-aminobenzoylglutamate was compromised, the animals were healthy and largely indistinguishable from control littermates. Several minor phenotypic changes in animals from one laboratory were reported but they were not present in the animals from the other laboratory. These animal models suggested that NAT1 was not required for normal development and survival, at least in the mouse, and did not point to any essential function for the protein.

Surprisingly, transgenic animals over-expressing human NAT1 showed a severe phenotype, with most animals not surviving to term [24]. Those that did survive had NAT1 activities that were only slightly higher than endogenous levels, suggesting that there is a strong selection process during fetal development for high NAT1 activity and points towards a need for NAT1 gene regulation *in vivo*. While mice can survive without the protein, they do not appear to tolerate its over-expression. This is an interesting observation that led us to examine the phenotypic changes induced in cancer cells with altered expression of the NAT1 protein.

We have shown that NAT1 knock-down inhibits cell proliferation by lowering the growth saturation density, which was accompanied by a decrease in cell viability. Morphological changes, anchorage-independent growth and E-cadherin expression supported speculation that NAT1 knock-down alters cell-cell contact inhibition. Our data indicated that off-target gene silencing is unlikely to account for the phenotypic changes. We also showed that up-regulation of E-cadherin was not confined to the HT-29 cell line. Up-regulation of E-cadherin mRNA suggested that NAT1 knock-down increased E-cadherin transcription, possibly by a change in DNA methylation status of the cells since levels of the transcriptional repressors Snail, Slug and Twist were not altered.

Loss of contact inhibition is a common trait in cancer [25] and restoration of this function is associated with expression of a family of growth-arrest-specific genes [26]. Cam and coworkers showed that NAT1 mRNA expression decreased by 57% in cancer cells following growth arrest induced by contact inhibition [27]. Moreover, when NAT1 was over-expressed in HB4a cells, they showed continued proliferation at cell densities where normal HB4a cell growth reached a plateau [8]. These observations support our current results that demonstrate a relationship between NAT1 expression and the ability for cells to undergo growth arrest. E-cadherin is believed to be beneficial in cancer treatment as this multi-faceted tumor suppressor protein is involved in many aspects of cancer biology including cell contact inhibition [28], [29], [30]. Experimental and clinical studies are in agreement that loss of E-cadherin is strongly associated with poor disease survivals [31], [32], [33], [34], [35], [36].

NAT1 knock-down significantly slowed tumor formation *in vivo*, although the effect was only marginal. This may, in part, be due to the loss of shRNA function *in vivo* since both NAT1 activity and mRNA levels were not suppressed to the same level as that observed *in vitro*. The tumors formed from cells with NAT1 knock-down appeared more differentiated suggesting that some phenotypic changes may remain *in vivo*. Nevertheless, others have shown that up-regulation of E-cadherin in cancer cells still allows tumor formation, albeit at a slower rate [37]. Perhaps of greater interest is the general loss of metastatic potential in cells that express E-cadherin [38]. This was not investigated in the present study because HT-29 cells do not readily metastasize in the nude mouse model following subcutaneous administration. We are currently investigating the effect of NAT1 knock-down in MDA-MB-231, a highly metastatic breast cancer cell line, in nude mice.

NAT1 catalyses the acetylation of arylamines via a cysteine-histidine-aspartic acid catalytic triad similar to that found in the transglutaminases and cysteine proteases [39]. The catalytic triad has also been implicated in the stability of NAT1 [5]. It remains unclear whether the NAT1 acetylating capacity is responsible for the observed phenotypic changes in the present study, or whether other mechanisms, such as protein-protein interaction, could account for these changes. We attempted to address this question by re-introducing a cysteine mutant of NAT1, which is catalytically inactive, into the shRNA 3.2 cells to rescue the phenotype. However, we were unable to isolate clones that expressed increased levels of NAT1 protein, possibly due to the instability of the mutant protein which is rapidly poly-ubiquitinated [5].

In conclusion, the current study has identified a phenotypic change in cancer cells associated with the loss of NAT1 protein. It shows that NAT1 may function beyond a Phase II xenobiotic metabolizing enzyme and raises the possibility that it is a potential therapeutic target in cancer. Further studies are required to map out the molecular mechanism underlying the cellular changes following NAT1 knock-down.
